# Cholesterol and breast cancer risk: a cohort study using health insurance claims and health checkup databases

**DOI:** 10.1007/s10549-023-06917-z

**Published:** 2023-03-30

**Authors:** Nobuhiro Narii, Ling Zha, Masayo Komatsu, Tetsuhisa Kitamura, Tomotaka Sobue, Toshio Ogawa

**Affiliations:** 1 Department of Social and Environmental Medicine, Graduate School of Medicine, Osaka University, Osaka, Japan; 2 Department of Food and Nutrition, Faculty of Agriculture, Setsunan University, Osaka, Japan; 3 Department of Environmental Medicine and Population Sciences, Graduate School of Medicine, Osaka University, 2-2 Yamadaoka, Suita, Osaka 565-0871 Japan

**Keywords:** Cholesterol, Triglyceride, Breast cancer, Japanese, Health insurance claims

## Abstract

**Purpose:**

This study aimed to investigate the association between serum cholesterol and triglyceride levels and breast cancer risk in Japanese women.

**Methods:**

We retrospectively evaluated the association between the levels of low-density lipoprotein cholesterol (LDL-C), high-density lipoprotein cholesterol (HDL-C), and triglycerides (TGs) and the incidence of breast cancer in a cohort study by using the health insurance claims and health checkup data from a database provided by JMDC Inc. We included 956,390 women who were insured between April 2008 and June 2019, identified breast cancer cases by using validated definitions, and estimated the risk of breast cancer by using multivariable Cox proportional hazards regression models adjusted for potential confounders.

**Results:**

During the 2,832,277 person-years observation period (median 2.4 years), 6284 participants were diagnosed with breast cancer. There was marginally significant association between LDL-C and breast cancer risk when comparing the highest and lowest quintiles and at the clinical cutoff values for diagnosing hyperlipidemia. HDL-C was not associated with breast cancer. However, when stratified by age groups (< 50 and ≥ 50), HDL-C was inversely associated with breast cancer risk in women over 50 years old. TG was not associated with breast cancer risk.

**Conclusion:**

In this population, there was a modest association of LDL-C at the clinical cutoff values for diagnosing hyperlipidemia (140 mg/mL), and there were no associations of HDL-C and TG with breast cancer risk.

**Supplementary Information:**

The online version contains supplementary material available at 10.1007/s10549-023-06917-z.

## Introduction

Breast cancer is the most common malignant disease worldwide, accounting for 24% of new cancer cases and 15% of cancer-related deaths in 2018 [1]. The incidence of breast cancer is also increasing in Japan. In 2018, it was the most common malignancy among women, with more than 90,000 new cases.

However, the role of cholesterol in cancer development remains controversial [2], and the association between cholesterol levels and breast cancer has not yielded consistent results. Comprehensive meta-analysis studies found an inverse association between high-density lipoprotein cholesterol (HDL-C) and breast cancer risk in postmenopausal or premenopausal women, but there was no association between low-density lipoprotein cholesterol (LDL-C) and breast cancer risk [3, 4]. On the other hand, Mendelian randomization (MR) studies reported that genetically elevated plasma HDL-C and LDL-C levels were positively associated with breast cancer risk [5–7], but some studies indicated that there was no association between genetically elevated plasma HDL-C and LDL-C levels and breast cancer risk [8]. Patients with dyslipidemia receive lipid-modifying agents such as 3-hydroxy-3-methylglutaryl coenzyme A reductase inhibitors (statins), but studies have not been able to exclude these effects. Furthermore, the distribution of risk factors for breast cancer and the incidence of breast cancer varies across countries [1, 9]; however, to the best of our knowledge, no large Japanese studies have comprehensively examined the association between cholesterol and breast cancer risk.

JMDC Inc. provides an epidemiological database that has accumulated claims and health checkup data from multiple health insurers in Japan, including a cumulative population of approximately 14 million people. In this study, we aimed to evaluate the association between cholesterol and breast cancer risk in Japanese women by using the database of health insurance claims and health checkup data.

## Materials and methods

### Data source and study participants

This study used the data of health insurance claims and health checkups collected by JMDC Inc., Tokyo, Japan. In Japan, all citizens are provided with a universal health insurance program, and each employer is required to provide its employees with insurance and regular health checkup opportunities. The JMDC Claims Database is an epidemiological claims database that has accumulated claims (inpatient, outpatient, and dispensing) and health checkup data from multiple health insurers since 2005. The cumulative population is approximately 14 million (as of February 2022), and the data enable the study of the prevalence and/or incidence rate of any disease in the general population, including healthy people, and can track hospital transfers or multiple facility visits. The JMDC Claims Database includes information on employee demographics, medical history, drug prescriptions, and hospital claims records based on International Classification of Diseases 10th Edition (ICD-10) coding.

We recruited 1,283,619 women who were insured between April 2008 and July 2019 and had at least one health checkup during that period. We excluded participants who had been insured for less than one year at the start of follow-up (*n* = 179,388) to ascertain cancer incidence and medication prescription status prior to the start of follow-up. We also excluded participants with no LDL-C, HDL-C, or triglyceride (TG) data (*n* = 29,499) and those with a breast cancer diagnosis prior to the start of follow-up (*n* = 4,523). To eliminate the influence of lipid-modifying agents, we excluded participants who had been prescribed lipid-modifying agents (WHO-ATC code: C10) at least once from 1 year before the start of follow-up to the end of follow-up (*n* = 114,050). The final analysis cohort consisted of 956,390 women.

### Cholesterol levels and other covariates

Age, body mass index (BMI), blood pressure, and fasting laboratory values, including cholesterol and blood glucose levels, were collected at the time of health checkup by using a standardized protocol at each health care institution. Data on smoking status, alcohol consumption, and physical activity were collected at the time of the health checkup by using a self-administered questionnaire. Hormonal medication use was defined using the insurance claims of drug prescriptions.

On the basis of the cholesterol level at the time of the initial health checkup (the start of follow-up), the participants were classified into quintiles, and the group with the lowest cholesterol level was used as the reference. The following definitions were used for the other covariates [10]. Hypertension was defined as a systolic blood pressure of 140 mmHg or higher, diastolic blood pressure of 90 mmHg or higher, or use of antihypertensive drugs. Diabetes mellitus was defined as fasting blood glucose of 125 or higher or use of diabetes medication. Current smokers were participants who have had a total of 100 or more cigarettes or had smoked for 6 months or longer and also smoked in the last month. Physical activity was defined as ≥ 30 min of exercise for more than 2 times a week or more than 1 h of walking per day. Current hormone users were participants who were prescribed hormone drugs (WHO-ATC code: G03C estrogens, G03D progestogens, or G03F progestogens and estrogens in combination) at least once from one year before the start of follow-up to the start of follow-up. There were no data on hormonal contraceptives for systemic use (WHO-ATC code; G03A) in the JMDC Claims Database because they are not covered by insurance in Japan.

### Case identification

Breast cancer was identified using an algorithm combining the diagnosis code for breast cancer (ICD-10 code: C500 to C506, C508, and C509), breast cancer–specific procedures, radiotherapy, and drugs. Details regarding breast cancer–specific procedures, radiotherapy, and drugs are reported elsewhere [11]. This algorithm has been shown to be a valid algorithm for identifying patients with newly diagnosed cancer from a Japanese claims database by comparison with the national cancer registry data [11]. In addition, other validation studies have been conducted in Japan to identify breast cancer by using a claims database, and the accuracy was reported to be high when the definition was combined disease codes and cancer treatment codes (surgery, chemotherapy, medication, and radiation procedure) [12, 13]. These studies also suggest that the Japanese claims database can accurately define the incidence of breast cancer.

The month of breast cancer incidence was defined as the month in which the ICD-10 codes for breast cancer, treatmen, and drugs were recorded in the claims in the same month during the insurance coverage period. The day of breast cancer incidence was defined as the 15th day of the month of breast cancer incidence.

### Statistical analysis

The baseline characteristics of the participants are presented using means and standard deviations or medians and interquartile ranges for continuous variables and percentages for categorical variables.

The Cox proportional hazards regression model was used to calculate hazard ratios (HRs) and 95% confidence intervals (CIs) to describe the risk of breast cancer incidence associated with LDL-C, HDL-C, or TG. Person-years of follow-up for each participant were calculated from the date of the first health checkup and censored at the date of breast cancer incidence, withdrawal from insurance due to death or job change, or end of the study period (July 31, 2019) whichever occurred first. On the basis of the LDL-C, HDL-C, or TG level at the time of the initial health checkup (the start of follow-up), the participants were classified into quintiles, and the group with the lowest LDL, HDL, or TG level was used as the reference. *P* values for trends were calculated by assigning scores for each quintile category as an independent continuous variable in the model. Alternatively, they were divided by the clinical cutoff values for diagnosing hyperlipidemia (140 mg/ml for LDL-C, 40 mg/ml for HDL-C, and 150 mg/ml for TG), and the group with the lowest value was used as the reference. The analysis was adjusted for known breast cancer risk factors as confounders [14]. Model 1 was stratified by age group (< 50 and ≥ 50) and adjusted for age (continuous). Model 2 was stratified by age groups (< 50 and ≥ 50) and adjusted for age (continuous), BMI (< 18.5, 18.5–25, 25–30, or > 30 kg/m^2^), hypertension (yes or no), diabetes mellitus (yes or no), current smoker (yes, no, or missing), drinking status (daily, sometimes, rarely, or missing), physical inactivity (yes or no), and current hormone use (yes or no).

A stratified analysis by age group (< 50 and ≥ 50 years) was conducted using age 50 as a surrogate indicator of menopausal status to examine effect modification by menopausal status. *P* values for interaction were calculated by adding product terms for LDL-C, HDL-C, or TG and age groups (< 50 and ≥ 50) to the main models, with adjustments for the aforementioned confounders. We conducted a sensitivity analysis that excluded participants with a follow-up period of less than 5 years to prevent potential reverse causation and ensure a sufficient latent period.

All *P* values reported were two-sided, and the significance level was set at *P* < 0.05. Statistical analyses were performed using Stata (version 16.0; Stata Corporation, College Station, TX, USA).

## Results

During the 2,832,277 person-years observation period (median: 2.4 years), 6284 participants were diagnosed with breast cancer. The crude incidence rate was 222 cases per 100,000 person-years. Table 1 shows the baseline characteristics of the study participants according to the quintile of LDL-C level at their first health checkup during the observation period. The highest LDL-C group had a higher average age and higher percentage of BMI (> 30), hypertension, diabetes, and physical activity than the lowest LDL-C group. However, the highest LDL-C group had a lower percentage of smokers, daily alcohol drinkers, and hormone users than the lowest LDL-C group. The observation period was slightly shorter for the participants with the highest LDL-C than those with the lowest LDL-C but did not differ substantially from the median (2.4 years) for all participants in either group.

**Table 1:** Baseline characteristics of the study population according to the quintile of LCL-C

	Quintile (LDL-C)				
	Q1	Q2	Q3	Q4	Q5
Number of subjects	198,711	185,216	190,628	197,808	184,027
LDL-C, median (IQR)	79 (71–85)	97 (94–101)	112 (108–116)	129 (124–134)	154 (146–167)
Age (years), mean (SD)	38.3 (9.3)	41 (9.7)	43.6 (10)	46.6 (10.2)	50 (9.9)
Body mass index, %					
< 18.5	24.0	19.3	15.9	12.4	8.3
18.5–25	70.4	71.6	71.4	69.8	66.2
25–30	4.6	7.3	9.9	13.7	19.2
> 30	0.9	1.6	2.7	3.9	5.9
Missing	0.2	0.2	0.2	0.3	0.4
Hypertension (yes), %	4.7	6.3	9.0	12.9	17.4
Diabetes (yes), %	0.5	0.7	1.1	1.5	2.3
Current smoker^a^, %					
Yes	11.3	9.5	9.2	9.4	9.6
No	78.5	81.8	83.4	84.4	85.4
Missing	10.2	8.7	7.4	6.3	5.0
Frequency of alcohol drinking, %					
Rarely	37.9	43.8	46.9	49.4	52.5
Occasionally	29.2	28.6	27.8	27.0	25.8
Daily	14.0	10.3	9.3	8.4	7.4
No information	18.8	17.3	16.1	15.2	14.3
Physical activity (yes)^b^, %	35.7	36.1	36.4	37.4	38.1
Current hormone drug user^c^, %	5.6	5.1	4.9	4.6	3.9
Follow-up period, median (IQR)	2.4 (1.0–4.6)	2.5 (1.1–4.7)	2.5 (1.1–4.6)	2.4 (1.1–4.5)	2.3 (1.0–3.8)

Continuous data are presented as mean (standard deviation) or median (interquartile range). Categorical variables are presented as percentages (%)

*LDL-C* low-density lipoprotein cholesterol, *IQR* interquartile range, *SD* standard deviation

^a^Participants who have had a total of 100 or more cigarettes or smoked for 6 months or longer and smoked in the last month

^b^Exercise of more than 2 times a week for 30 min or more or more than 1 h of walking per day

^c^Participants who were prescribed hormone drugs at least once from 1 year before the start of follow-up to the start of follow-up

Table 2 shows the HRs of incidence for breast cancer according to the quintile of LDL-C, HDL-C, and TG. Model 2 showed an increased risk of breast cancer in the highest LDL-C group compared with the lowest LDL-C group [HR_Q1 vs. Q5_ = 1.08 (95% CI 0.99–1.18), *P*-trend = 0.273] with marginal statistical significance. In an analysis at the clinical cutoff values for diagnosing hyperlipidemia (140 mg/ml), the risk of breast cancer was also increased [HR_<140 mg/ml vs. ≥140 mg/ml_ = 1.09 (95% CI 1.02–1.16)]. There was no association between HDL-C or TG and breast cancer in either quintile or the clinical cutoff values for diagnosing hyperlipidemia [HR_Q1 vs. Q5_ = 0.98 (95% CI 0.90–1.06), *P*-trend = 0.307; HR_<40 mg/dl vs. ≥40 mg/dl_ = 1.22 (95% CI 0.91–1.65) for HDL-C; HR_Q1 vs. Q5_ = 1.02 (95% CI 0.94–1.11), *P*-trend = 0.745; HR_<150 mg/dl vs. ≥150 mg/dl_ = 0.99 (95% CI 0.89–1.10) for TG].

**Table 2:** HRs of the incidence for breast cancer according to the quintile of blood cholesterol and triglycerides

Quintile: median (IQR)^a^	Cases	IR^b^	Model 1		Model 2	
			HR	95% CI	HR	95% CI
LDL-C (mg/dl)						
Q1: 79 (71–85)	1087	179	Ref		Ref	
Q2: 97 (94–101)	1207	211	1.05	0.97–1.14	1.06	0.97–1.15
Q3: 112 (108–116)	1270	219	0.99	0.92–1.08	1.00	0.92–1.09
Q4: 129 (124–134)	1318	226	0.94	0.86–1.02	0.94	0.87–1.02
Q5: 154 (146–167)	1402	285	1.07	0.99–1.17	1.08	0.99–1.18
*P*-trend			0.250		0.273	
< 140 mg/dl	4882	209	Ref			
≥ 140 mg/dl	1402	285	1.09	1.02–1.15	1.09	1.02–1.16
HDL-C (mg/dl)						
Q1: 53 (48–56)	1258	220	Ref		Ref	
Q2: 63 (61–65)	1347	219	1.02	0.94–1.10	1.01	0.94–1.09
Q3: 71 (69–73)	1102	214	0.99	0.91–1.07	0.98	0.91–1.07
Q4: 79 (77–82)	1288	224	1.01	0.93–1.09	1.00	0.93–1.09
Q5: 93 (88–100)	1289	232	0.99	0.91–1.07	0.98	0.90–1.06
*P*-trend			0.361		0.307	
< 40 mg/dl	44	189	Ref			
≥ 40 mg/dl	6240	222	1.22	0.91–1.65	1.22	0.91–1.65
Triglycerides (mg/dl)						
Q1: 39 (34–42)	1089	185	Ref		Ref	
Q2: 51 (48–54)	1206	212	1.04	0.96–1.13	1.03	0.95–1.12
Q3: 64 (61–68)	1319	226	1.04	0.96–1.13	1.04	0.95–1.12
Q4: 83 (77–90)	1358	238	1.04	0.96–1.12	1.03	0.95–1.11
Q5: 127 (110–158)	1312	251	1.03	0.95–1.12	1.02	0.94–1.11
*P*-trend			0.933		0.745	
< 150 mg/dl	5903	220	Ref			
≥ 150 mg/dl	381	253	1.00	0.90–1.11	0.99	0.89–1.10

Model 1 was stratified by age groups (< 50 and ≥ 50) and adjusted for age (continuous)

Model 2 was stratified by age group (< 50 and ≥ 50) and adjusted for age (continuous), body mass index (< 18.5, 18.5–25, 25–30, or > 30 kg/m^2^), hypertension (yes or no), diabetes mellitus (yes or no), current smoker (yes, no, or missing), drinking status (daily, sometime, rarely, or missing), physical inactivity (yes or no), and current hormone use (yes or no)

*IQR* interquartile range, *IR* incidence rates, *CI* confidence interval, *HDL-C* high-density lipoprotein cholesterol, *HR* hazard ratio, *LDL-C* low-density lipoprotein cholesterol, *Ref* reference

^a^In each quintile category, values of blood cholesterol and triglycerides are presented as median (*IQR* interquartile range)

^b^Incidence rates per 100,000 person-years

Lipid abnormalities may be present even before cancer is detected because they are the most prominent metabolic abnormalities in cancer [15]. In addition, breast cancer requires several years to develop [16]. To prevent potential reverse causation and ensure a sufficient latent period, we performed sensitivity analysis only on participants with an observation period of at least five years (Table S1). The results showed a positive association between LDL-C and breast cancer risk, similar to that in the main analysis. The HR estimate was slightly larger than in the main analysis and was statistically significant [HR_Q1 vs. Q5_ = 1.32 (95% CI 1.03–1.69), *P*-trend = 0.185; HR_<140 mg/ml vs. ≥140 mg/ml_ = 1.24 (95% CI 1.02–1.50)]. There were no differences in HDL-C and TG levels, similar to that in the main analysis.

Breast cancer has distinct causes and prognoses in patients of premenopausal and postmenopausal age [1]. To examine the differences in the effects of LDL-C, HDL-C, and TG on the risk of breast cancer between premenopausal and postmenopausal women, we performed a stratified analysis. Given that there were no data on menopausal status in the JMDC Claims Database, we referred to the average age of menopause in Japan as 50 years [1, 17] and treated participants under and over 50 years old as premenopausal and postmenopausal, respectively. The crude incidence rate was lower in women under 50 years of age (197 cases per 100,000 person-years) than in women over 50 years of age (297 cases per 100,000 person-years). In women under 50 years old, the highest LDL-C group had increased breast cancer risk [HR _Q1 vs. Q5_ = 1.13 (95% CI 1.02–1.25), *P*-trend = 0.715] compared with the lowest LDL-C group, whereas there was no association in women over 50 years old [HR _Q1 vs. Q5_ = 0.94 (95% CI 0.79–1.12), *P*-trend = 0.469] (Table 3). Furthermore, the risk of breast cancer was significantly increased in an analysis using the clinical cutoff values for diagnosing hyperlipidemia [HR _<140 mg/ml vs. ≥140 mg/ml_ = 1.15 (95% CI 1.06–1.26)] in women under 50 years old, but there was no difference in women over 50 years old [HR _<140 mg/ml vs. ≥140 mg/ml_ = 1.04 (95% CI 0.94–1.14)] (Table 3). The interaction of age group (< 50 and ≥ 50 years) was statistically significant in both analyses (*P*-interaction = 0.017 and 0.008). In a sensitivity analysis with a five-year latent period (Table S2), there was an increased risk of breast cancer incidence in women under 50 years old [HR _Q1 vs. Q5_ = 1.39 (95% CI 1.06–1.82), *P*-trend = 0.077; HR _<140 mg/ml vs. ≥140 mg/ml_ = 1.31 (95% CI 1.05–1.64)]. No difference was found in women over 50 years of age (Table S2).

**Table 3:** HRs of the incidence for breast cancer according to the quintile of blood cholesterol and triglycerides by age groups (< 50 and ≥ 50)

Quintile: median (IQR)^a^	Women under 50 years (*n* = 679,531)				Women over 50 years (*n* = 265,620)				
	Cases	IR^b^	HR^c^	95% CI	Cases	IR^b^	HR^c^	95% CI	*P*-interaction^d^
LDL-C (mg/dl)									
Q1: 79 (71–85)	931	167	Ref		156	314	Ref		
Q2: 97 (94–101)	958	195	1.04	0.95–1.13	249	309	0.98	0.80–1.19	
Q3: 112 (108–116)	901	200	0.98	0.89–1.07	369	286	0.90	0.74–1.08	
Q4: 129 (124–134)	774	199	0.91	0.82–1.00	544	280	0.87	0.72–1.04	
Q5: 154 (146–167)	659	260	1.13	1.02–1.25	743	310	0.94	0.79–1.12	
*P*-trend			0.715				0.469		0.017
< 140 mg/dl	3564	189	Ref		1318	291			
> 140 mg/dl	659	260	1.15	1.06–1.26	743	310	1.04	0.95–1.14	0.008
HDL-C (mg/dl)									
Q1: 53 (48–56)	828	186	Ref		430	339	Ref		
Q2: 63 (61–65)	950	196	1.03	0.94–1.13	397	301	0.91	0.80–1.05	
Q3: 71 (69–73)	746	187	0.95	0.86–1.05	356	311	0.96	0.83–1.11	
Q4: 79 (77–82)	882	204	0.99	0.89–1.09	406	287	0.90	0.78–1.03	
Q5: 93 (88–100)	817	217	0.96	0.87–1.06	472	265	0.84	0.73–0.97	
*P*-trend			0.268				0.025		0.058
< 40 mg/dl	33	186	Ref		11	200			
> 40 mg/dl	4190	198	1.00	0.71–1.41	2050	298	1.63	0.90–2.95	0.227
Triglycerides (mg/dl)									
Q1: 39 (34–42)	888	174	Ref		201	265	Ref		
Q2: 51 (48–54)	922	200	1.04	0.94–1.14	284	266	0.99	0.83–1.19	
Q3: 64 (61–68)	903	204	1.01	0.92–1.11	416	294	1.08	0.92–1.28	
Q4: 83 (77–90)	843	213	1.01	0.92–1.12	515	297	1.08	0.91–1.27	
Q5: 127 (110–158)	667	203	0.98	0.88–1.09	645	330	1.15	0.98–1.35	
*P*-trend			0.639				0.033		0.292
< 150 mg/dl	4049	198	Ref		1854	293			
> 150 mg/dl	174	191	0.93	0.79–1.08	207	349	1.10	0.95–1.28	0.050

*IQR* interquartile range, *IR* incidence rate, *CI* confidence interval, *HDL-C* high-density lipoprotein cholesterol, *HR* hazard ratio, *LDL-C* low-density lipoprotein cholesterol, *Ref* reference

^a^In each quintile category, values of blood cholesterol and triglycerides are presented as median (interquartile range)

^b^Incidence rates per 100,000 person-years

^c^HR was calculated using the Cox proportional hazard model (Model 2), which was adjusted for age (continuous), body mass index (< 18.5, 18.5–25, 25–30, or > 30 kg/m^2^), hypertension (yes or no), diabetes mellitus (yes or no), current smoker (yes, no, or missing), drinking status (daily, sometime, rarely, or missing), physical inactivity (yes or no), and current hormone use (yes or no)

^d^*P*-interaction is interaction by age group (< 50 and ≥ 50)

In the stratified analysis of age groups (< 50 and ≥ 50) (Table 3), there was a significant inverse association between HDL-C and breast cancer in women over 50 years old [HR _Q1 vs. Q5_ = 0.84 (95% CI 0.73–0.97), *P*-trend = 0.025]; however, there was no association in women under 50 years old [HR _Q1 vs. Q5_ = 0.96 (95% CI 0.87–1.06), *P*-trend = 0.268], with a marginally statistically significant interaction of age groups (< 50 and ≥ 50). In a sensitivity analysis with a five-year latent period (Table S2), the reduction in the risk of breast cancer incidence in women over 50 years old was attenuated in terms of statistical significance [HR _Q1 vs. Q5_ = 0.76 (95% CI 0.45–1.30), *P*-trend = 0.833], but the effect estimates remained similar compared with the main analysis. The analysis at the clinical cutoff values for diagnosing hyperlipidemia showed no difference in either group, and there were wide CIs for the estimates because of the very small number of breast cancer cases among participants with HDL-C < 40 mg/ml (Table 3).

In the stratified analysis of age groups (< 50 and ≥ 50) (Table 3), there was some evidence of a positive association between TG and breast cancer in women over 50 years [HR _Q1 vs. Q5_ = 1.15 (95% CI 0.98–1.35), *P*-trend = 0.033, *P*-interaction = 0.292]. However, there was no statistically significant difference when the lowest TG group was compared with the highest TG group, and there was no statistically significant interaction by age group. There was no association between TG and breast cancer in women under 50 years [HR = 0.98 (95% CI 0.88–1.09), *P*-trend = 0.639]. Sensitivity analysis with a five-year latent period (Table S2) showed an increase in the point estimate of the HR, but there was no statistically significant difference in women over 50 years [HR _Q1 vs. Q5_ = 1.63 (95% CI 0.75–3.56), *P*-trend = 0.598]. The analysis of the clinical cutoff values for diagnosing hyperlipidemia showed no difference in either group (Table 3).

## Discussion

This cohort study analyzed the health insurance claims and health checkup data of more than 950,000 women in Japan and found that women with LDL-C ≥ 140 mg/ml had a modest but significantly increased breast cancer risk compared with women with LDL-C < 140 mg/ml.

This study showed a modestly positive association between LDL-C and breast cancer risk [HR _Q1 vs. Q5_ = 1.08 (95% CI 0.99–1.18), *P*-trend = 0.273; HR _<140 mg/ml vs. ≥140 mg/ml_ = 1.09 (95% CI 1.02–1.16)]. Previous meta-analyses of observational studies have found no association between LDL-C and breast cancer risk [3, 4]. They were conducted mainly in Europe and America, with few Japanese data. The limitation of these studies is that the effects of lipid-modifying agents, including statins, which significantly alter serum LDL-C levels, have not been ruled out. A recent MR study reported that genetically elevated LDL-C increases the risk of breast cancer [5–7]. Proliferating cancer cells have an increased requirement for cholesterol, thus increasing the expression of the LDL-C receptor and uptake of LDL-C into breast cancer tissues [18]. The metabolites of lipid peroxide cause conformational changes in deoxyribonucleic acid (DNA) and reduce DNA repair capacity. Delimaris et al. [19] reported that breast cancer patients have elevated serum levels of oxidized LDL-C and that serum levels of oxidized LDL-C are associated with increased breast cancer risk. Given these findings, the results obtained in the present study indicate that LDL-C increases breast cancer risk.

Statins reduce serum LDL-C. A meta-analysis examining the association between statins and breast cancer risk did not find an association between statins and breast cancer risk, although there was significant heterogeneity between the estimates [20]. However, stratified analyses showed that statins have some breast cancer preventive effect in Asians compared with Americans and in long-term statin users compared with short-term statin users. Breast cancer rates vary widely by country [1, 9]; therefore, studies of the association between LDL-C, statins, and breast cancer may need to be restricted to Asians, including the Japanese.

In this study, no association was found between serum HDL-C levels and the risk of breast cancer. However, among women over 50 years of age, those with higher HDL-C levels may have a lower risk of breast cancer. Previous meta-analyses have reported that higher HDL-C levels decreases the risk of postmenopausal breast cancer [3, 4], and this finding is similar to that of the present study. HDL-C has antioxidant and anti-inflammatory effects, inhibits the LDL-C oxidation cascade. Furthermore, apolipoprotein A-I, which is the main component of HDL-C in serum, inhibits cell proliferation [18]. These mechanisms suggest that HDL suppresses breast cancer development. The differential effect of HDL-C on breast cancer risk depending on menopausal status is difficult to interpret. Ni et al. [3] suggested that low HDL-C indicates relative androgen deficiency and that breast cancer risk in postmenopausal women may be explained by the association between androgens and breast cancer risk. By contrast, a large population-based cohort study in Japan showed that subjects with HDL-C ≥ 40 mg had an increased risk of breast cancer compared with subjects with HDL-C ≤ 40 mg [21]. In the present study, participants with HDL-C < 40 mg/ml accounted for 0.87% (*n* = 8341) of the total participants (n = 956,390). The small number of breast cancer cases (*n* = 44) limited the statistical power of the analysis at the clinical cutoff values for diagnosing hyperlipidemia.

In this study, no association was found between TG and risk of breast cancer, although there was some evidence of a positive association between TG and breast cancer in women over 50 years [HR _Q1 vs. Q5_ = 1.15 (95% CI 0.98–1.35), *P*-trend = 0.033, *P*-interaction = 0.292]. Previous reports showed that there was no association between TG and breast cancer [5], but others showed an inverse association between TG and breast cancer [3, 8]. Studies on the mechanisms linking TG and breast cancer are scarce. Therefore, the association between TG levels and breast cancer needs to be further investigated in detail.

This study has several strengths. First, the analysis was based on a large individual-traceable database. Second, the outcome (breast cancer incidence) was identified by a highly validated definition from health insurance claims data. Third, the analysis excluded individuals taking lipid-modifying agents, such as statins, thus allowing for the estimation of effects that exclude the influence of these drugs. In other words, the analysis was conducted in a population whose exposure (cholesterol or triglyceride levels) was relatively stable during the observation period. This study had several limitations. First, important risk factors for breast cancer (e.g., age at menarche, genetic factors, family history of breast cancer, fat intake, parturition, lactation status, and contraception) [14] may have confounded the effect of cholesterol. These factors were not available in the JMDC Claims Database and could not be adjusted in the multivariable analysis. Second, breast cancer cannot be divided into subtypes according to receptor expression status. Given that nonclinical studies have shown that the effect of LDL-C and HDL-C on breast cancer development is dependent on receptor expression status [18], the effect of cholesterol on breast cancer may vary by subtype even in a clinical setting. Third, the observation period for this study was relatively short (median: 2.4 years), considering that breast cancer has a long-term induction and latency period. However, a sensitivity analysis restricted to participants with an observation period > 5 years yielded results with a similar trend.

In conclusion, this study showed the modest association of LDL-C at the clinical cutoff values for diagnosing hyperlipidemia (140 mg/mL) and indicated that HDL-C and TG had no associations with breast cancer risk in Japanese women.

## Supplementary Information

Below is the link to the electronic supplementary material.

Supplementary file1 (DOCX 21 KB)

Supplementary file2 (DOCX 23 KB)

## Data Availability

The database used in this study is available for purchase from JMDC Inc., Tokyo, Japan (https://www.jmdc.co.jp/en/).
