# Supplementary material for: Cholesterol and breast cancer risk: a cohort study using health insurance claims and health checkup databases
Source: Breast Cancer Res Treat. 2023 Mar 30;199(2):315–22. doi: 10.1007/s10549-023-06917-z (PMC10175375; doi:10.1007/s10549-023-06917-z)
Supplement: Supplementary file 1 — Supplementary file1 (DOCX 21 KB) [file 10549_2023_6917_MOESM1_ESM.docx]

**Table S1. HRs of the incidence for breast cancer according to the quintile of blood cholesterol and triglycerides with a five-year latent period**

|  |  |  |  | Model 1 | |  | Model 2 | |
| --- | --- | --- | --- | --- | --- | --- | --- | --- |
|  | Quintile: Median (IQR)^a^ | Cases | IR^b^ | HR | 95% CI |  | HR | 95% CI |
| LDL-C (mg/dl) | |  |  |  |  |  |  |  |
|  | Q1: 79 (71–85) | 161 | 188 | Ref. |  |  | Ref. |  |
|  | Q2: 97 (94–101) | 175 | 225 | 1.10 | 0.88–1.36 |  | 1.12 | 0.90–1.38 |
|  | Q3: 112 (108–116) | 165 | 224 | 1.03 | 0.83–1.28 |  | 1.05 | 0.84–1.31 |
|  | Q4: 129 (124–134) | 161 | 243 | 1.06 | 0.85–1.33 |  | 1.09 | 0.87–1.37 |
|  | Q5: 154 (146–167) | 134 | 306 | 1.28 | 1.01–1.63 |  | 1.32 | 1.03–1.69 |
|  | *P*-trend |  |  | 0.260 |  |  | 0.185 |  |
|  | <140 mg/dl | 662 | 218 | Ref. |  |  |  |  |
|  | ≥140 mg/dl | 134 | 306 | 1.22 | 1.01–1.48 |  | 1.24 | 1.02–1.50 |
| HDL-C (mg/dl) | |  |  |  |  |  |  |  |
|  | Q1: 53 (48–56) | 169 | 254 | Ref. |  |  | Ref. |  |
|  | Q2: 63 (61–65) | 158 | 210 | 0.84 | 0.67–1.04 |  | 0.84 | 0.67–1.04 |
|  | Q3: 71 (69–73) | 145 | 226 | 0.89 | 0.71–1.11 |  | 0.89 | 0.71–1.11 |
|  | Q4: 79 (77–82) | 155 | 214 | 0.83 | 0.66–1.03 |  | 0.82 | 0.65–1.03 |
|  | Q5: 93 (88–100) | 169 | 246 | 0.91 | 0.73–1.12 |  | 0.89 | 0.71–1.11 |
|  | *P*-trend |  |  | 0.396 |  |  | 0.331 |  |
|  | <40 mg/dl | 3 | 112 | Ref. |  |  |  |  |
|  | ≥40 mg/dl | 793 | 230 | 2.07 | 0.67–6.43 |  | 2.15 | 0.69–6.70 |
| Triglycerides (mg/dl) | |  |  |  |  |  |  |  |
|  | Q1: 39 (34–42) | 154 | 200 | Ref. |  |  | Ref. |  |
|  | Q2: 51 (48–54) | 181 | 247 | 1.16 | 0.93–1.43 |  | 1.16 | 0.93–1.43 |
|  | Q3: 64 (61–68) | 161 | 219 | 0.99 | 0.79–1.24 |  | 0.99 | 0.79–1.23 |
|  | Q4: 83 (77–90) | 176 | 259 | 1.13 | 0.91–1.40 |  | 1.11 | 0.89–1.39 |
|  | Q5: 127 (110–158) | 124 | 223 | 0.96 | 0.75–1.22 |  | 0.93 | 0.72–1.19 |
|  | *P*-trend |  |  | 0.560 |  |  | 0.429 |  |
|  | <150 mg/dl | 763 | 230 | Ref. |  |  |  |  |
|  | ≥150 mg/dl | 33 | 222 | 0.91 | 0.64–1.29 |  | 0.87 | 0.61–1.24 |

a: In each quintile category, values of blood cholesterol and triglycerides are presented as median (interquartile range).

b: Incidence rates per 100,000 person-years.

Model 1 was stratified by age groups (<50 and ≥50) and adjusted for age (continuous).

Model 2, stratified by age group (<50 and ≥50), was adjusted for age (continuous), body mass index (<18.5, 18.5–25, 25–30, or >30 kg/m^2^), hypertension (yes or no), diabetes mellitus (yes or no), current smoker (yes, no, or missing), drinking status (daily, sometime, rarely, or missing), physical inactivity (yes or no), and current hormone use (yes or no).

Abbreviations: IQR, interquartile range; IR, incidence rates; CI, confidence interval; HDL-C, high-density lipoprotein cholesterol; HR, hazard ratio; LDL-C, low-density lipoprotein cholesterol; Ref, reference.
