# Supplementary material for: Cholesterol and breast cancer risk: a cohort study using health insurance claims and health checkup databases
Source: Breast Cancer Res Treat. 2023 Mar 30;199(2):315–22. doi: 10.1007/s10549-023-06917-z (PMC10175375; doi:10.1007/s10549-023-06917-z)
Supplement: Supplementary file 2 — Supplementary file2 (DOCX 23 KB) [file 10549_2023_6917_MOESM2_ESM.docx]

**Table S2. HRs of the incidence for breast cancer according to the quintile of blood cholesterol and triglycerides by age groups (<50 and ≥50) with a five-year latent period**

|  |  | Women under 50 years  (n = 679,531) | | | |  | Women over 50 years  (n = 265,620) | | | |  |
| --- | --- | --- | --- | --- | --- | --- | --- | --- | --- | --- | --- |
|  | Quintile: Median (IQR)^a^ | Cases | IR^b^ | HR^c^ | 95% CI |  | Cases | IR^b^ | HR^c^ | 95% CI | *P*-interaction^d^ |
| LDL-C (mg/dl) | |  |  |  |  |  |  |  |  |  |  |
|  | Q1: 79 (71–85) | 153 | 187 | Ref. |  |  | 8 | 204 | Ref. |  |  |
|  | Q2: 97 (94–101) | 156 | 218 | 1.09 | 0.87–1.36 |  | 19 | 301 | 1.45 | 0.63–3.32 |  |
|  | Q3: 112 (108–116) | 146 | 228 | 1.08 | 0.86–1.37 |  | 19 | 194 | 0.91 | 0.39–2.08 |  |
|  | Q4: 129 (124–134) | 121 | 232 | 1.06 | 0.83–1.35 |  | 40 | 285 | 1.29 | 0.60–2.78 |  |
|  | Q5: 154 (146–167) | 92 | 310 | 1.39 | 1.06–1.82 |  | 42 | 297 | 1.29 | 0.60–2.80 |  |
|  | *P*-trend |  |  | 0.077 |  |  |  |  | 0.585 |  | 0.593 |
|  | <140 mg/dl | 576 | 214 | Ref. |  |  | 86 | 253 |  |  |  |
|  | >140 mg/dl | 92 | 310 | 1.31 | 1.05–1.64 |  | 42 | 297 | 1.10 | 0.75–1.60 | 0.549 |
| HDL-C (mg/dl) | |  |  |  |  |  |  |  |  |  |  |
|  | Q1: 53 (48–56) | 138 | 236 | Ref. |  |  | 31 | 382 | Ref. |  |  |
|  | Q2: 63 (61–65) | 145 | 220 | 0.91 | 0.72–1.16 |  | 13 | 142 | 0.40 | 0.21–0.78 |  |
|  | Q3: 71 (69–73) | 122 | 218 | 0.88 | 0.68–1.13 |  | 23 | 280 | 0.85 | 0.49–1.47 |  |
|  | Q4: 79 (77–82) | 124 | 198 | 0.77 | 0.60–0.99 |  | 31 | 314 | 0.98 | 0.58–1.65 |  |
|  | Q5: 93 (88–100) | 139 | 248 | 0.90 | 0.70–1.15 |  | 30 | 234 | 0.76 | 0.45–1.30 |  |
|  | *P*-trend |  |  | 0.199 |  |  |  |  | 0.833 |  | 0.048 |
|  | <40 mg/dl | 3 | 131 | Ref. |  |  | 0 | -^e^ | -^e^ | -^e^ |  |
|  | >40 mg/dl | 665 | 224 | 1.66 | 0.53–5.20 |  | 128 | 268 | -^e^ | -^e^ | -^e^ |
| Triglycerides (mg/dl) | |  |  |  |  |  |  |  |  |  |  |
|  | Q1: 39 (34–42) | 146 | 204 | Ref. |  |  | 8 | 148 | Ref. |  |  |
|  | Q2: 51 (48–54) | 158 | 241 | 1.10 | 0.88–1.38 |  | 23 | 301 | 1.99 | 0.89–4.45 |  |
|  | Q3: 64 (61–68) | 136 | 215 | 0.96 | 0.76–1.21 |  | 25 | 245 | 1.56 | 0.70–3.47 |  |
|  | Q4: 83 (77–90) | 141 | 253 | 1.09 | 0.86–1.38 |  | 35 | 286 | 1.74 | 0.80–3.78 |  |
|  | Q5: 127 (110–158) | 87 | 202 | 0.88 | 0.66–1.16 |  | 37 | 292 | 1.63 | 0.75–3.56 |  |
|  | *P*-trend |  |  | 0.496 |  |  |  |  | 0.598 |  | 0.515 |
|  | <150 mg/dl | 651 | 226 | Ref. |  |  | 112 | 251 |  |  |  |
|  | >150 mg/dl | 17 | 151 | 0.64 | 0.39–1.04 |  | 16 | 445 | 1.51 | 0.88–2.59 | 0.007 |

a: In each quintile category, values of blood cholesterol and triglycerides are presented as median (interquartile range).

b: Incidence rates per 100,000 person-years.

c HR was calculated using the Cox proportional hazard model (Model 2), which was adjusted for age (continuous), body mass index (<18.5, 18.5–25, 25–30, or >30 kg/m^2^), hypertension (yes or no), diabetes mellitus (yes or no), current smoker (yes, no, or missing), drinking status (daily, sometime, rarely, or missing), physical inactivity (yes or no), and current hormone use (yes or no).

d: *P*-interaction is interaction by age group (<50 and ≥50)

e: IR, HR, and *P*-interaction were not calculated because there were no breast cancer cases in postmenopausal women over 50 years with HDL < 40 mg/dl.

Abbreviations: IQR, interquartile range; IR, incidence rate; CI, confidence interval; HDL-C, high-density lipoprotein cholesterol; HR, hazard ratio; LDL-C, low-density lipoprotein cholesterol; Ref, reference.
